# GLP-1 Receptor Agonists in Epilepsy: Separating Evidence for Antiseizure Activity, Neuroprotection, and Disease Modification

**DOI:** 10.3390/ijms27188036

**Published:** 2026-09-09

**Authors:** Yuliy A. Gorgul, Aleksey V. Zaitsev

**Affiliations:** Sechenov Institute of Evolutionary Physiology and Biochemistry, Russian Academy of Sciences, 194223 Saint-Petersburg, Russia; gorgul.yuliy@gmail.com

**Keywords:** GLP-1 receptor agonists, epilepsy, antiseizure activity, epileptogenesis, disease modification, neuroprotection, semaglutide, mechanistic causality

## Abstract

GLP-1 receptor (GLP-1R) agonists are increasingly investigated in epilepsy, but antiseizure activity, neuroprotection, and disease modification are distinct therapeutic claims. This critical narrative review with structured evidence mapping separates these claims across 21 preclinical primary publications and eight human studies identified through 6 August 2026. Selected GLP-1R-related interventions show antiseizure and anti-kindling signals, but effects vary across compounds, models, treatment timing, and seizure types; null and pro-seizure findings in absence epilepsy preclude a uniform class-wide antiseizure effect, and concurrent anti-kindling does not establish antiepileptogenesis. Neuroprotective evidence is broader, although direct neuronal or tissue preservation is demonstrated only in selected studies; many findings remain biomarker-based, and seizure reduction may itself lessen downstream injury. Evidence for durable disease modification remains suggestive rather than established. Causal support is strongest at the receptor level, whereas most downstream synaptic, inflammatory, glial, oxidative, and mitochondrial evidence remains associative. Semaglutide has high translational relevance but remains directly under-tested in epilepsy, and human evidence is predominantly observational or safety-oriented and does not establish therapeutic epilepsy efficacy. Progress requires chronic epilepsy studies with longitudinal EEG/video-EEG and baseline seizure burden, post-insult designs controlling initial-insult severity and assessing persistence after withdrawal, and linked pharmacokinetic, target-engagement, and causal mechanistic testing.

## 1. Introduction

Epilepsy is a disease of the brain characterized by an enduring predisposition to generate epileptic seizures, but seizure control remains incomplete for a substantial proportion of patients despite multiple antiseizure medications [1,2]. Current pharmacotherapy is predominantly directed toward suppressing seizures, whereas clinically established approaches that reliably prevent epileptogenesis or durably modify the underlying disease course remain limited [3]. Epileptogenesis broadly refers to the processes through which epilepsy develops and may subsequently progress; in this review, prevention or delay of epilepsy development and persistent modification of established epilepsy are nevertheless treated as distinct therapeutic questions [4]. Reduction in seizure frequency during treatment therefore remains conceptually distinct from modification of the processes that generate or sustain epilepsy.

Glucagon-like peptide-1 receptor (GLP-1R) agonists were developed primarily for metabolic indications and are now widely used in diabetes and obesity care [5]. GLP-1R is a class B1 G-protein-coupled receptor that couples predominantly to Gαs, activating adenylyl cyclase and increasing intracellular cAMP, with protein kinase A (PKA) and exchange proteins directly activated by cAMP (Epac) among its principal downstream effectors; additional signaling pathways may be engaged depending on cellular context [6]. Their neurological relevance grew from evidence that GLP-1R-related therapies can influence CNS-associated biology, together with broader preclinical observations involving neuronal injury, inflammatory responses, synaptic regulation, and cellular metabolism [7]. Several of these processes, particularly neuroinflammation, glial responses, and cellular/metabolic remodeling, are themselves implicated in epileptogenesis [8]. These observations prompted investigation of GLP-1R signaling in seizure and epilepsy models.

Epilepsy-specific investigation has expanded from receptor-level and acute provoked-seizure studies to kindling and repeated-provocation paradigms, post-status epilepticus interventions, and selected chronic and genetic epilepsy models [9,10,11]. More recently, observational and safety-oriented human studies have examined seizure- and epilepsy-related outcomes in large clinical datasets [12,13]. Epilepsy is heterogeneous across seizure types, epilepsy types, syndromes, and etiologies [14]. This heterogeneity is directly relevant to interpretation of the GLP-1R literature: null and pro-seizure findings in absence-epilepsy models caution against generalizing positive results from convulsive paradigms to epilepsy as a whole [11,15].

Favorable findings in this literature can support three non-equivalent therapeutic claims: antiseizure activity, neuroprotection, and antiepileptogenic or disease-modifying effects. Seizure suppression during active treatment is not, by itself, evidence of disease modification; neuroprotection alone is insufficient to demonstrate altered epileptogenesis; and concurrent anti-kindling should not be equated with prevention of epilepsy [16,17]. Treatment-associated changes in inflammatory, synaptic, glial, or cellular-stress markers may likewise be biologically informative without identifying a causal mechanism unless receptor-, pathway-, or cell-specific intervention provides evidence for that link.

A second source of overinterpretation is pharmacological heterogeneity. “GLP-1R-related interventions” encompasses selective GLP-1R agonists, native GLP-1 and engineered analogues, dual or multi-receptor agonists, and adjacent incretin-based strategies such as DPP-4 inhibition [5,18]. Results obtained with one intervention cannot automatically be transferred to another compound, and effects of multi-receptor agonists cannot be assigned specifically to GLP-1R without receptor-level dissection. This is especially important for semaglutide. Its widespread clinical use gives it high translational relevance, but its direct epilepsy-specific evidence remains substantially narrower than that for some other GLP-1R-related compounds [5,19]. Within the literature identified for this review through 6 August 2026, no randomized therapeutic epilepsy trial tested a GLP-1R agonist using prospectively defined seizure-frequency or electrographic efficacy endpoints.

This critical narrative review uses structured evidence mapping and organizes the literature by therapeutic claim rather than by molecular pathway. Evidence for antiseizure effects, neuroprotection, and antiepileptogenic or disease-modifying activity is evaluated separately, followed by assessment of receptor dependence and downstream mechanistic causality. Drug- and receptor-specific boundaries are preserved across individual agonists, semaglutide, multi-receptor therapies, and adjacent incretin strategies, while emerging human data are considered in a compact translational context. We also identify experiments needed to resolve the major remaining gaps. The central question is not whether GLP-1R-related interventions produce favorable biological changes, but what therapeutic claim each experiment actually supports. This ordering keeps seizure suppression, neuronal protection, and alteration of disease course distinct before considering mechanism and clinical translation.

## 2. Literature Identification and Evidence Interpretation

This review was conducted as a critical narrative review with structured evidence mapping rather than as a formal systematic review. Literature searches were conducted using Scopus and Google Scholar through 6 August 2026. Search queries combined GLP-1/GLP-1R terminology and individual intervention names, including liraglutide, semaglutide, exenatide/exendin-4, and related incretin therapies, with seizure- and epilepsy-related terms such as seizure, epilepsy, convulsion, kindling, pentylenetetrazole/PTZ, kainate/kainic acid, pilocarpine, status epilepticus, and febrile seizure. Citation tracking and examination of related publications were subsequently used for targeted verification and contextual evidence. Direct preclinical mapping prioritized primary in vivo seizure or epilepsy studies reporting seizure-related outcomes or direct assessment of seizure-associated injury; reviews, duplicate reports, and in vitro-only studies were not counted among the direct therapeutic preclinical publications, although mechanistic studies were retained where relevant to interpretation. Human studies were retained when seizure-, epilepsy-, or relevant neurological safety outcomes were reported for GLP-1R-related therapies. At the locked literature cutoff, the structured preclinical evidence map comprised 21 primary publications and the human translational evidence set comprised eight studies.

Evidence directness was assessed claim by claim according to whether the experimental design actually addressed the therapeutic or mechanistic inference under evaluation. Relevant features included the intervention tested, model and disease state, treatment timing, whether seizure outcomes were provoked or spontaneous, the type of endpoint, and whether effects were assessed only during active exposure or persisted after treatment withdrawal. These distinctions determine, for example, whether an acute pretreatment experiment supports altered seizure susceptibility rather than treatment of established epilepsy, whether concurrent kindling demonstrates anti-kindling activity rather than antiepileptogenesis, and whether post-SE molecular changes provide direct evidence for disease modification. Direct neuronal or tissue-preservation endpoints were likewise distinguished from surrogate molecular markers, and receptor or pathway perturbation from downstream biomarker association.

Methodological confidence was considered independently and reflected how reliably the selected design supported the inference it was intended to test. This appraisal considered adequacy of controls, experimental unit and sample flow, randomization and blinding where applicable and reported, completeness and reliability of seizure ascertainment, adequacy of recording or observation for the selected endpoint, attrition, statistical reporting, possible pseudoreplication, and reporting inconsistencies. A feature could affect both dimensions in different ways: for a chronic epilepsy claim, for example, whether spontaneous seizures were assessed is primarily a question of directness, whereas the completeness and duration of that seizure assessment affect confidence in the resulting inference. Methodological confidence therefore remained claim-specific and was not converted into a single overall study-quality rating. The direct preclinical evidence map is summarized in Table 1.

Targeted integrity checks included examination of primary papers and their associated supplementary material, source and DOI verification, corrections, retractions or expressions of concern, and obvious reporting or image concerns where encountered. Related publications were considered when assessing whether apparently separate evidence was independent or shared substantial experimental lineage; papers from the same research group were not automatically treated as duplicates. Resolvable dose-unit discrepancies were recorded as reporting errors rather than automatic exclusion criteria, and null, discordant, and pro-seizure findings were retained. The “Key interpretive consideration” entries in Table 1 summarize the design features and inferential boundaries most relevant to individual claims rather than collapsing claim-specific methodological confidence into a single study-level rating. Mechanistic claims were additionally distinguished as association, receptor dependence, pathway intervention, or cell-specific causality, so biomarker changes were not treated as causal evidence by default. Human evidence was evaluated separately because it is predominantly observational, safety-oriented, or pharmacovigilance-based rather than derived from randomized therapeutic epilepsy trials.

## 3. Conceptual and Pharmacological Framework

### 3.1. Distinct Therapeutic Claims

**Figure 1:**
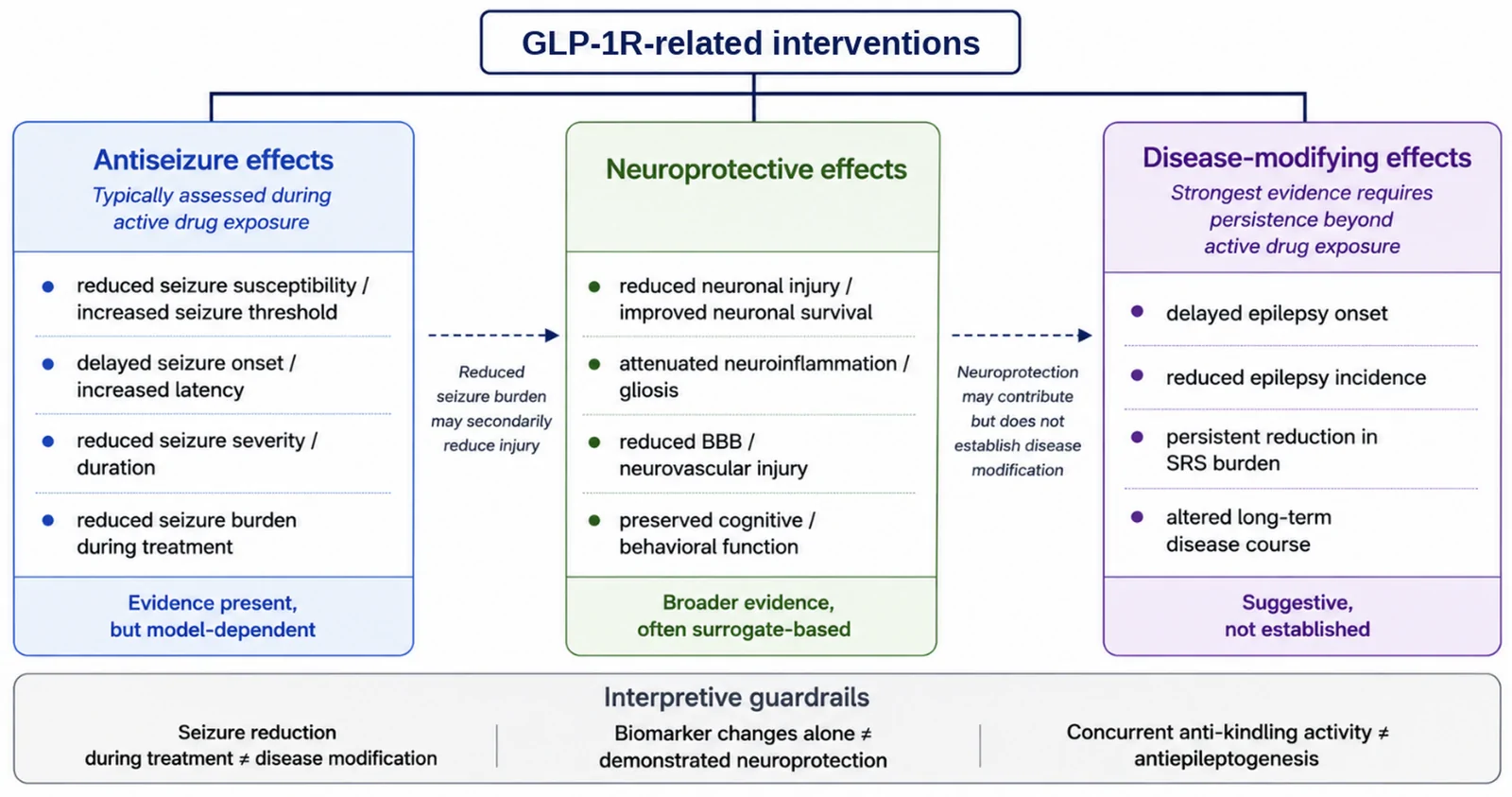
Antiseizure, neuroprotective, and disease-modifying effects represent distinct therapeutic claims in the evaluation of GLP-1R-related interventions in epilepsy. GLP-1R-related therapies may reduce seizure susceptibility or seizure burden, attenuate tissue injury and associated pathological changes, or modify the long-term course of epilepsy. These outcomes are biologically related but not interchangeable. Acute seizure suppression may secondarily reduce injury-related biomarkers, and neuroprotection may be compatible with, but does not by itself establish, disease modification. The strongest evidence for disease-modifying effects requires post-insult intervention, assessment of spontaneous seizures, prolonged EEG/video-EEG follow-up, and ideally persistence after treatment withdrawal. Abbreviations: BBB, blood–brain barrier; GLP-1R, glucagon-like peptide-1 receptor; SRS, spontaneous recurrent seizures.

Whether a favorable experimental outcome supports an antiseizure, neuroprotective, or disease-modifying claim depends on the endpoint measured, treatment timing, whether epilepsy is already established, and whether the effect persists beyond active drug exposure. These three domains are therefore treated here as related but non-interchangeable therapeutic claims (Figure 1) [16,17].

An antiseizure effect is defined operationally in this review as a direct reduction in seizure expression or susceptibility under the conditions tested. Outcomes used for this assessment include seizure threshold or latency, behavioral seizure severity, seizure duration, electrographic seizure activity, and spontaneous recurrent seizure frequency or burden. Treatment timing is integral to interpretation. Acute pretreatment can demonstrate altered susceptibility to a provoked seizure but does not test treatment of established epilepsy. Attenuation of kindling during active treatment likewise supports anti-kindling activity rather than prevention of epilepsy [16,17]. Seizure suppression during treatment is therefore interpreted separately from antiepileptogenesis.

Neuroprotection is assessed separately and, in this review, refers primarily to preservation of neurons or brain tissue from seizure- or epilepsy-associated injury. Greatest weight is given to neuronal survival, quantified neurodegeneration, or other sufficiently direct measures of tissue damage. Changes in cytokines, oxidative-stress indices, apoptosis-related proteins, autophagy markers, or mitochondrial-dynamics-associated proteins can support a neuroprotective interpretation but are not equivalent to demonstrated neuronal or tissue preservation. The relationship with antiseizure effects is also asymmetric. If an intervention reduces the severity or duration of the seizure insult, subsequent reductions in inflammation, cellular stress, or neuronal injury may partly reflect the smaller insult itself. Neuronal preservation could in turn contribute to improved long-term outcome, but neuroprotection alone is insufficient to demonstrate disease modification.

An antiepileptogenic or disease-modifying effect requires evidence that treatment alters the development, persistence, or subsequent course of epilepsy rather than merely suppressing seizures while pharmacological exposure continues. Within this broader domain, prevention or delay of epilepsy development after an epileptogenic insult and persistent modification of already established epilepsy are distinct therapeutic questions. The strongest support comes from designs incorporating appropriately timed post-insult or established-epilepsy treatment, spontaneous recurrent seizures or epilepsy incidence, longitudinal EEG or video-EEG, and assessment after treatment withdrawal [17]. In post-insult designs, the severity of the initiating insult should be adequately documented and, where possible, balanced or controlled, because differences in later epilepsy burden may otherwise reflect differences in initial injury rather than modification of downstream epileptogenesis [17]. Persistence beyond active exposure is particularly informative because it helps distinguish durable alteration of disease course from residual or ongoing antiseizure pharmacology. When these elements are incomplete, calibrated terms such as suggestive evidence, candidate disease-modifying effect, or consistent with disease modification are more appropriate than claiming established antiepileptogenesis.

### 3.2. Pharmacological Boundaries of GLP-1R-Related Evidence

Pharmacological generalization poses a separate interpretive problem even when the therapeutic claims are correctly distinguished. In this review, “GLP-1R-related interventions” is an organizational umbrella, not a claim that all included compounds have equivalent pharmacology, exposure, or therapeutic effects.

Selective GLP-1R agonists themselves differ in molecular structure, duration of exposure, protein binding, pharmacokinetics, and tissue distribution [5,36]. These differences can materially affect both the magnitude and the anatomical site of receptor engagement. Consequently, an effect observed with one GLP-1R agonist should not automatically be generalized to another compound or to the entire class. This distinction is particularly important for semaglutide. Findings obtained with liraglutide, exendin-4, or other GLP-1R agonists can provide biological context and support hypothesis generation, but they do not constitute direct evidence for semaglutide efficacy. Semaglutide-specific conclusions must rest on semaglutide-specific experiments.

Native GLP-1 and engineered GLP-1 analogues are likewise informative for GLP-1R biology but are not pharmacologically equivalent to approved long-acting agonists [5,36]. Their effects should therefore be attributed to the intervention actually tested rather than transferred across compounds.

An even stricter boundary applies to dual- and multi-receptor agonists. Effects produced by compounds acting at both GLP-1R and GIPR, or at additional incretin-related receptors, cannot be assigned specifically to GLP-1R unless receptor-level dissection demonstrates such dependence. Receptor multiplicity may increase therapeutic interest while simultaneously weakening mechanistic attribution. DPP-4 inhibitors occupy a related but distinct category: by increasing endogenous incretin signaling they provide adjacent incretin-based evidence, but they are not direct GLP-1R agonists and should not be treated as pharmacologically equivalent [5,18].

Accordingly, the following sections evaluate GLP-1R-related interventions primarily by therapeutic claim while preserving drug-specific, receptor-specific, model-specific, and treatment-timing boundaries. This structure is necessary to prevent favorable findings in one experimental context from being converted into broader claims that the available evidence does not support.

## 4. Antiseizure Evidence

Direct antiseizure signals have been reported for several GLP-1R-related interventions, but the evidence is heterogeneous across drugs, seizure paradigms, and treatment schedules. Most positive studies assessed seizure susceptibility or severity during pretreatment or concurrent drug exposure rather than treatment initiated after a documented baseline of established epilepsy, and important null and pro-seizure findings preclude a class-wide interpretation. The direct preclinical evidence is summarized in Table 1. The relevant question is therefore not whether GLP-1R activation is uniformly “anticonvulsant,” but under which experimental conditions a given intervention reduces seizure expression and how closely those conditions approximate treatment of ongoing epilepsy.

### 4.1. Acute Provoked Seizure Models

Acute provocation studies show that selected GLP-1R agonists can alter seizure susceptibility while the intervention is present, but they provide little information about efficacy in established epilepsy. Exendin-4 pretreatment reduced acute behavioral seizure severity after kainic acid administration, whereas exenatide reduced penicillin-induced cortical spike frequency during selected recording intervals without significantly changing spike latency or amplitude [28,29]. Liraglutide also showed acute antiseizure activity in distinct convulsant paradigms: pretreatment before pentylenetetrazole reduced electrographic activity and behavioral seizure scores and increased latency to myoclonic jerks, while pretreatment before picrotoxin delayed convulsion onset and shortened tonic convulsions [30,31]. Because treatment preceded seizure induction, these findings demonstrate acute modulation of seizure susceptibility during drug exposure rather than therapeutic suppression of recurrent seizures in established epilepsy.

The acute literature is not uniformly positive. Intracerebroventricular native GLP-1 did not significantly alter the incidence, latency, or behavioral severity of pilocarpine-induced status epilepticus [27]. This null result argues against treating native GLP-1, exendin-derived compounds, and long-acting clinical agonists as pharmacologically interchangeable.

Receptor-manipulation studies provide complementary evidence for GLP-1R involvement but are distinct from agonist-efficacy studies. In an acute kainate paradigm, *Glp1r* loss worsened seizures in a gene-dose-dependent pattern, whereas hippocampal rescue improved the phenotype [9]. In vivo pharmacological GLP-1R inhibition with exendin(9–39) likewise increased behavioral and electrographic seizure measures in a febrile-seizure paradigm [32]. These findings support receptor-dependent modulation of seizure susceptibility in specific models, but they do not establish antiseizure efficacy of approved systemic GLP-1R agonists in chronic epilepsy.

### 4.2. Kindling and Repeated-Provocation Paradigms

Repeated-provocation studies extend beyond single acute challenges, but interpretation depends critically on whether treatment is present throughout kindling. Wen et al. provide the strongest receptor-level evidence in this group: liraglutide delayed progression to severe PTZ-kindled seizures, reduced seizure measures, and these principal effects were reversed by exendin(9–39) [10]. A challenge approximately four days after treatment also suggested residual attenuation. Because this post-treatment assessment remained a PTZ provocation within a repeated-provocation paradigm and follow-up was short, it provides limited evidence that the anti-kindling effect outlasted active treatment but is insufficient to establish a durable treatment-independent alteration of epileptogenesis.

Semaglutide has direct epilepsy-specific evidence from a PTZ-kindling study in which treatment began before kindling and continued during acquisition; the final PTZ/ECoG challenge also followed a further semaglutide dose [19]. Semaglutide reduced selected seizure scores, the proportion of fully kindled animals, and final seizure duration. This supports semaglutide anti-kindling activity under active treatment, not an off-treatment effect or modification of the subsequent disease course.

Other liraglutide studies point in the same general direction but add more limited evidentiary weight. The PTZ- and corneal-kindling studies by Koshal and Kumar reported reduced behavioral seizure severity during concurrent treatment and should be regarded as related studies rather than independent replications [23,24]. Hussein et al. similarly reported behavioral attenuation of PTZ kindling without withdrawal assessment [25]. De Souza et al. is especially informative for interpretation: liraglutide delayed progression to stage 5 PTZ kindling but did not provide a seizure advantage over levetiracetam, supporting delayed kindling rather than prevention of epilepsy [26]. Across these studies, attenuation during repeated provocation is evidence of anti-kindling activity; concurrent treatment alone does not establish antiepileptogenesis.

### 4.3. Chronic, Spontaneous, and Genetic Seizure Models

Studies using spontaneous recurrent seizures or genetically determined seizure phenotypes are more relevant to chronic disease than acute or repeatedly provoked models, but they remain few and generally lack treatment initiation after a documented pretreatment seizure baseline. The kainate arm of Citraro et al. provides the strongest chronic SRS signal: liraglutide reduced observed SRS burden and cumulative seizure duration on video-EEG [11]. However, there was no pretreatment baseline seizure burden, EEG sampling was limited, and treatment began early after kainate. The study therefore supports an antiseizure signal using chronic SRS outcomes, while questions of disease modification require separate analysis.

Zhang et al. reported an additional post-SE SRS signal with exendin-4, including reduced SRS frequency and duration [21]. This finding is low-confidence because seizure-monitoring details and experimental units were incompletely described. It should therefore be treated as supportive evidence using chronic SRS outcomes, without inferring durable modification of epilepsy.

In a *Scn1a*+/− Dravet model, liraglutide was associated with reduced seizure susceptibility and reported EEG abnormalities [20]. The limited longitudinal quantitative seizure assessment makes this evidence hypothesis-generating rather than a strong demonstration of sustained seizure control. In genetically epilepsy-prone rats, liraglutide showed little acute antiseizure activity but modest benefit during chronic treatment and attenuated diazepam tolerance, preserving benzodiazepine responsiveness [22]. This result addresses modulation of diazepam responsiveness rather than broad synergy between GLP-1R agonists and antiseizure medications.

### 4.4. Absence Epilepsy and Seizure-Type Specificity

Absence epilepsy provides the clearest counterexample to a uniform antiseizure interpretation. In the WAG/Rij arm of Citraro et al., prolonged liraglutide treatment did not prevent the age-dependent development of spike-wave discharges. After a 30-day withdrawal period, quantitative analysis based on three 3 h EEG sessions in six animals per group provided no evidence of SWD prevention [11]. This study therefore does not test suppression of already established absence seizures and should not be interpreted as such.

In established absence epilepsy, exendin-4 produced the opposite direction of effect. A single dose increased both the number and duration of spike-wave discharges in WAG/Rij rats, while discharge amplitude was unchanged [15]. The 180 min ECoG window and possible effects of vigilance or behavioral state limit interpretation but do not negate the observed proabsence signal. Together, these findings show that positive results in convulsive models cannot be generalized to absence epilepsy.

Overall, direct antiseizure evidence for GLP-1R-related interventions is present but heterogeneous and strongly dependent on drug, model, seizure type, and treatment timing. Acute pretreatment and concurrent-kindling studies provide the largest body of positive evidence, whereas studies using chronic SRS outcomes are fewer, with Citraro et al. providing the strongest positive signal and Zhang et al. offering lower-confidence support. Crucially, these SRS studies were not initiated after a documented baseline of established epilepsy. The absence-epilepsy evidence is discordant with the predominantly positive convulsive-model literature: liraglutide did not prevent SWD development, and exendin-4 increased SWD burden. No uniform antiseizure GLP-1R agonist class effect is therefore established.

## 5. Neuroprotective Evidence

Neuroprotective findings recur more often across the preclinical literature than evidence for disease modification, but their strength depends heavily on the endpoint used. As summarized in Table 1, only a subset of studies directly assessed neuronal survival or tissue injury; many others relied predominantly on molecular, inflammatory, or cell-stress surrogates. Their interpretation must also remain separate from seizure suppression. A reduction in the severity of the initiating seizure insult may itself lessen subsequent tissue damage, whereas neuronal preservation, even when demonstrated directly, does not by itself show that the long-term course of epilepsy has been modified.

### 5.1. Direct Evidence of Neuronal or Tissue Protection

Direct measures of neuronal injury or survival provide the clearest neuroprotective evidence. During et al. are particularly informative in the acute kainate paradigm because *Glp1r* loss worsened both the seizure phenotype and neuronal injury, whereas hippocampal receptor rescue improved the phenotype [9]. The loss-and-rescue design supports receptor-dependent protection against kainate-associated neuronal injury in this experimental setting. Its scope is nonetheless narrow: it neither tests a clinically used GLP-1R agonist nor addresses protection in chronic epilepsy, although it provides stronger causal leverage for GLP-1R involvement than pharmacological studies based solely on treatment-associated biomarkers.

Direct histological protection has also been reported with liraglutide and exendin-4. In the kainate/SRS arm of Citraro et al., liraglutide was associated with partial preservation of CA1 pyramidal neurons [11]. The chronic seizure context gives this result particular relevance, but liraglutide simultaneously reduced the measured seizure burden. Consequently, neuronal preservation cannot be assumed to represent a seizure-independent protective action; reduced recurrent seizure exposure may have contributed to the histological difference.

Acute pretreatment presents an even clearer attribution problem. Exendin-4 administered before kainate reduced hippocampal neuronal death together with acute seizure severity and inflammatory and blood–brain barrier-related endpoints [28]. Because neuronal death was assessed directly, the study provides a genuine tissue-protection signal. Yet treatment preceded the insult and also attenuated seizures, so some of the subsequent reduction in injury may reflect a milder effective seizure insult. The BBB, inflammatory, and Lcn2 findings characterize a broader treatment-associated response but do not identify the mechanism responsible for neuronal preservation.

Other studies provide less direct support. In a post-status epilepticus model, exendin-4 treatment was accompanied by reduced apoptosis-related and reactive-glial/inflammatory readouts [21]. These findings are compatible with protection, but the neuroprotective inference is less direct than in studies centered on quantified neuronal survival, and cell-specific GLP-1R dependence was not established.

### 5.2. Neuroprotective Signals in Early Post-SE Studies

Early post-SE studies help classify tissue and cellular outcomes separately from antiseizure effects because several did not include post-treatment seizure endpoints. The same design feature limits mechanistic attribution, however: without seizure monitoring, it is not possible to determine whether post-treatment seizure activity contributed to the observed tissue changes or whether the apparent protection was seizure-independent.

Wang et al. reported that liraglutide administered after lithium–pilocarpine status epilepticus shifted inflammatory and apoptosis-related markers toward a less injury-associated profile [33]. However, direct neuronal survival was not demonstrated. The maximum justified inference is therefore that treatment produced molecular changes compatible with reduced cellular stress or injury, not that liraglutide prevented neuronal death.

Antmen et al. similarly initiated liraglutide treatment after status epilepticus and reported changes in redox-, inflammatory-, and mitochondrial-dynamics-associated markers, together with limited behavioral evidence of functional protection [34]. These observations broaden the range of treatment-associated injury signals but do not demonstrate restored mitochondrial function or brain bioenergetic rescue. No post-treatment seizure endpoint was included, further restricting the study to early post-SE tissue and behavioral outcomes.

Tian et al. provide relatively direct supportive evidence because treatment with DA3-CH after diazepam-terminated status epilepticus was associated with NeuN preservation together with changes in glial, cytokine, and apoptosis-related markers [35]. However, DA3-CH is a dual GLP-1R/GIPR agonist, so this protection cannot be attributed specifically to GLP-1R activation. In addition, equivalence of initial status epilepticus severity between groups was not demonstrated, limiting attribution of subsequent tissue preservation specifically to treatment. Follow-up was limited to seven days and no chronic seizure outcome was assessed. The study therefore supports early post-SE neuroprotective activity of a dual incretin agonist rather than selective GLP-1R-mediated disease modification.

### 5.3. Biomarker-Heavy and Functionally Ambiguous Evidence

A substantial additional body of literature reports protective-looking molecular changes without comparably direct tissue endpoints. In the Dravet model, liraglutide was associated with reported neuronal preservation as well as reduced apoptosis-related markers [20]. The neuronal-preservation finding provides a direct supportive neuroprotective endpoint, whereas the apoptosis-related measures remain surrogate evidence. Methodological limitations constrain the weight that can be assigned to the overall neuroprotective inference.

During semaglutide treatment in PTZ kindling, hippocampal injury-associated measures and cognition improved alongside reduced seizure severity [19]. Here, the concurrent antiseizure effect provides an important alternative explanation for at least part of the downstream tissue and behavioral benefit. Similarly, redox-, apoptosis-, and cell-stress-associated changes reported in liraglutide kindling studies, including Hussein et al., should be treated as supportive biological signals rather than direct demonstrations of neuronal preservation.

Across selected seizure and post-SE paradigms, GLP-1R-related interventions repeatedly show signals of neuronal or tissue protection, but confidence varies with endpoint directness. Studies measuring neuronal injury or survival directly are the most informative; much of the remaining literature consists of molecular, inflammatory, redox, or cell-stress surrogates. In pretreatment and concurrent-treatment designs, lower seizure severity may itself account for part of the subsequent tissue benefit. The aggregate evidence for neuroprotection is stronger than that for durable disease modification, although seizure-independent protection is not uniformly established and most proposed downstream mechanisms remain associative. Neuronal preservation may be relevant to the later disease course without constituting evidence of disease modification.

## 6. Antiepileptogenic and Disease-Modifying Evidence

Disease-modifying or antiepileptogenic claims require evidence beyond seizure suppression during active treatment or attenuation of seizure-associated tissue injury. In post-insult paradigms, the most informative designs initiate treatment after a defined epileptogenic insult, document or control the severity of that insult, follow spontaneous recurrent seizures (SRS) or epilepsy incidence longitudinally, and ideally assess persistence after treatment withdrawal [17]. When treatment begins after epilepsy is established, a pretreatment seizure baseline is similarly important for separating treatment effects from baseline disease severity. Figure 1 captures this distinction between on-treatment benefit and alteration of disease course. Against this standard, the current GLP-1R-related literature offers suggestive rather than definitive disease-modifying evidence; Table 1 shows how few studies incorporate chronic/SRS outcomes compared with kindling, acute, or early post-SE designs.

### 6.1. Chronic and Spontaneous-Seizure Studies: Suggestive Signals, but No Definitive Demonstration

The kainate/SRS arm of Citraro et al. provides the strongest candidate disease-modifying signal in the current literature. Liraglutide treatment was initiated early after kainate; spontaneous recurrent seizures were subsequently assessed, and treatment was associated with reduced measured SRS burden and cumulative seizure duration together with partial CA1 neuronal preservation [11]. The inclusion of spontaneous seizures makes this design substantially more informative for disease-course inference than acute provocation or concurrent-kindling studies, and a limited off-treatment assessment provides some evidence relevant to persistence.

The disease-modifying interpretation of Citraro et al. is limited by several features of the design. Treatment began soon after the initiating insult rather than after epilepsy had been demonstrably established, no pretreatment seizure baseline was available, video-EEG sampling was restricted rather than continuous or prolonged, and persistence beyond treatment was assessed only briefly. These features leave unresolved whether the lower SRS burden reflects altered development of epilepsy or continued or recently discontinued pharmacological seizure suppression. The result is best described as a candidate disease-modifying effect consistent with, but not demonstrating, modification of epileptogenesis.

A second post-SE study reported lower SRS frequency and duration during exendin-4 treatment after pilocarpine-induced status epilepticus [21]. The use of spontaneous seizures gives this study greater disease-course relevance than kindling paradigms, but seizure monitoring was limited; there was no pretreatment SRS baseline, and no meaningful withdrawal or persistence assessment was included. Thus, it provides supportive chronic/SRS evidence but does not establish disease modification.

The Dravet syndrome study by Liu et al. raises a different question. Liraglutide was associated with reduced seizure susceptibility and reported EEG abnormalities, and later testing has been interpreted as suggesting persistence after treatment [20]. Quantitative longitudinal seizure assessment was limited, however, and methodological confidence for a durable disease-course inference is low. A genetic developmental epilepsy also differs conceptually from acquired post-insult epileptogenesis. The latter signal is therefore best regarded as hypothesis-generating evidence of a persistent effect rather than a demonstration of disease modification.

The developmental literature also contains a negative result. In WAG/Rij rats, prolonged liraglutide treatment did not prevent age-dependent spike-wave discharge development, with the preventive outcome assessed after treatment withdrawal [11]. This finding argues against a uniform class-wide preventive effect without excluding disease-modifying potential in other epilepsy types.

### 6.2. Anti-Kindling Activity Is Not Equivalent to Antiepileptogenesis

Kindling models probe progressive changes in seizure susceptibility induced by repeated provocation, but delayed kindling during concurrent treatment is an anti-kindling outcome and does not itself demonstrate antiepileptogenesis. In all included chemical and electrical kindling studies, GLP-1R-related treatment began before or during acquisition; none initiated treatment only after full kindling had been established, and prolonged off-treatment observation was generally absent. In PTZ paradigms, slower acquisition may partly reflect acute pharmacological modification of the chemoconvulsant-evoked seizure response rather than durable change in kindling-related plasticity. Electrical kindling avoids this chemoconvulsant-specific concern, although concurrent antiseizure action can still affect acquisition. These studies therefore support anti-kindling activity under or shortly after treatment but do not establish durable treatment-independent modification of epileptogenesis.

Wen et al. provide the most informative exception within this group. Liraglutide attenuated PTZ-kindling progression, exendin(9–39) reversed key effects, and a short challenge after treatment cessation suggested some residual anti-kindling activity [10]. The antagonist experiment strengthens receptor-level attribution, and the off-treatment challenge is more informative than concurrent treatment alone. However, the post-treatment assessment remained a PTZ challenge within the repeated-provocation paradigm, and persistence was examined only over a short interval. The study therefore supports receptor-dependent anti-kindling with limited evidence that the effect outlasted active treatment, but it does not establish durable antiepileptogenesis.

The same boundary is particularly important for semaglutide. Wang et al. showed that semaglutide reduced PTZ-kindling severity during concurrent treatment [19]. The final PTZ/ECoG challenge followed a further semaglutide dose, so there was no off-treatment seizure assessment. The study therefore provides direct semaglutide anti-kindling evidence under active exposure but cannot establish persistence after withdrawal, prevention of epilepsy, or disease modification. The related liraglutide studies by Koshal and Kumar and Hussein et al. likewise support anti-kindling activity without adding durable disease-course evidence.

De Souza et al. illustrate the distinction especially clearly: liraglutide delayed progression to stage 5 kindling, yet animals ultimately became fully kindled [26]. Delayed kindling under concurrent treatment is an anti-kindling effect and is not, by itself, evidence of epilepsy prevention or antiepileptogenesis.

### 6.3. Post-Insult Neuroprotection Does Not Establish Altered Disease Course

Early post-SE studies provide biologically relevant evidence but cannot substitute tissue or molecular outcomes for longitudinal epilepsy endpoints. Liraglutide treatment after lithium–pilocarpine SE produced inflammatory and apoptosis-related marker changes in Wang et al. and redox-, inflammatory-, and mitochondrial-dynamics-associated changes in Antmen et al. [33,34]. Neither study assessed the subsequent chronic epilepsy course with an appropriate SRS endpoint.

Tian et al. reported NeuN preservation together with glial, cytokine, and cell-survival-associated changes after treatment with DA3-CH following diazepam-terminated SE [35]. NeuN preservation provides supportive neuroprotective evidence, but follow-up lasted only seven days and chronic seizures were not assessed. Moreover, DA3-CH is a dual GLP-1R/GIPR agonist, so its effects cannot be assigned specifically to GLP-1R signaling. Equivalence of initial SE severity between groups was also not demonstrated, limiting attribution of subsequent tissue differences specifically to treatment.

More generally, initial-insult confounding can arise in two distinct ways. Treatment may itself alter the severity or consequences of the initiating insult, such that later differences in disease course partly reflect a milder initial injury. Alternatively, initial insult severity may simply be inadequately controlled, leaving equivalence between groups uncertain. This distinction matters because the severity of the initiating SE can influence the extent of acute injury and the substrate from which subsequent epilepsy develops; groups exposed to different initial insult burdens may therefore develop different chronic outcomes even without independent modification of downstream epileptogenic processes [17]. In either case, later differences in SRS burden or other disease-course outcomes cannot be attributed cleanly to downstream modification of epileptogenic processes. Neuronal preservation may be biologically compatible with disease modification, but without appropriate longitudinal seizure outcomes and persistence beyond treatment, it does not establish one.

Within the preclinical evidence set reviewed here, no study provides a definitive demonstration of antiepileptogenesis or durable disease modification by a GLP-1R-related intervention. Citraro et al. remain the strongest candidate signal, whereas the other chronic, kindling, and early post-SE findings do not provide comparable evidence of durable alteration of disease course. The present evidence is therefore suggestive, not evidence of established antiepileptogenesis.

## 7. Mechanistic Evidence: From Association to Causality

The mechanistic literature is extensive, but its causal resolution is much more limited than the number of reported molecular changes might suggest. Evidence is clearest at the level of GLP-1R itself; proposed downstream synaptic, inflammatory, glial, oxidative, mitochondrial, and cell-survival mechanisms are supported mainly by treatment-associated changes rather than direct pathway intervention. Table 2 captures this gradient from receptor-level causal evidence through limited functional support to a much larger body of associative biomarker data. Receptor dependence resolves one step in the causal chain, but it does not identify the intracellular pathway or cell type responsible for the therapeutic phenotype.

### 7.1. GLP-1R Dependence: The Strongest Causal Evidence

Receptor-level perturbation provides the clearest causal evidence. Although GLP-1R canonically signals through Gαs–cAMP–PKA/Epac and can engage additional context-dependent downstream pathways, epilepsy-specific studies have rarely tested whether individual intracellular branches are necessary for antiseizure or neuroprotective effects [6]. In the acute kainate paradigm, *Glp1r* loss worsened seizure severity and neuronal injury, heterozygous animals showed an intermediate phenotype, and hippocampal receptor rescue improved the outcome [9]. The combination of loss of function, gene-dose dependence, and rescue provides unusually strong evidence that GLP-1R signaling influences susceptibility to kainate-induced seizures and injury in this model. The inference remains paradigm-specific: it neither demonstrates efficacy of a clinically used GLP-1R agonist in chronic epilepsy nor identifies the neuronal, glial, vascular, or intracellular pathway downstream of the receptor.

Pharmacological receptor dependence is also supported in PTZ kindling. Wen et al. found that exendin(9–39) reversed key anti-kindling effects of liraglutide, supporting GLP-1R-dependent liraglutide action in that paradigm [10]. This provides stronger causal leverage than accompanying changes in receptor proteins or neurotransmitter concentrations, but the antagonist experiment does not establish that altered excitation–inhibition balance, inflammatory signaling, or another downstream pathway mediates seizure attenuation.

Zhang et al. provide additional receptor-level evidence in a febrile-seizure-related paradigm. In vivo pharmacological GLP-1R inhibition with exendin(9–39) worsened acute behavioral and electrographic seizure measures, including seizure severity, stage-4 bursts, EEG discharge amplitude, and seizure latency [32]. Complementary experiments in cultured hippocampal neurons showed that exendin(9–39) increased spontaneous burst firing and reduced sIPSC amplitude and frequency, while GLP-1R knockdown similarly reduced sIPSC amplitude and frequency. These findings support a role for GLP-1R signaling in maintaining inhibitory synaptic transmission, but the siRNA experiments did not demonstrate an in vivo seizure or EEG phenotype. GLP-1R knockdown also increased TRPV1 abundance and capsaicin-evoked current, identifying TRPV1 as a downstream candidate; however, TRPV1 necessity for the synaptic or seizure phenotype was not demonstrated.

### 7.2. Synaptic Excitation–Inhibition: Functional Evidence Is Limited

Synaptic regulation is often proposed as a direct explanation for GLP-1R-related antiseizure effects, but the functional evidence remains limited. The cultured-neuron findings from Zhang et al. constitute functional evidence that GLP-1R signaling can influence inhibitory synaptic transmission and are mechanistically more informative than measurements of neurotransmitter content or receptor abundance alone.

Evidence that GLP-1R agonists restore pathological excitation–inhibition balance is much less direct. In PTZ kindling, liraglutide altered expression profiles of GABA_A_, AMPA, and NMDA receptor-related proteins [10], while two linked liraglutide kindling studies reported treatment-associated changes in bulk brain GABA and glutamate concentrations [23,24]. These findings indicate treatment-associated remodeling but do not directly quantify the balance of excitatory and inhibitory synaptic currents in the epileptic network. Bulk postmortem transmitter concentrations in particular cannot establish functional synaptic E/I balance, and the observed synaptic changes have not been shown to be necessary for the in vivo antiseizure effect. The available functional data support GLP-1R regulation of inhibitory transmission, not restoration of pathological network E/I balance.

### 7.3. Neuroinflammatory and NLRP3-Related Signaling: Recurrent Association, Limited Causality

Inflammatory changes are among the most repeatedly reported findings after GLP-1R-related treatment. Reductions in NLRP3-associated readouts, cytokines, and other inflammatory markers have been described during semaglutide treatment in PTZ kindling and in several liraglutide, exendin-4, and incretin-related post-SE studies [19,21,34,35]. This recurrence provides biological plausibility for an anti-inflammatory contribution to the observed therapeutic phenotypes.

Inflammatory pathway mediation has not been tested with comparable rigor. Reductions in NLRP3 or downstream cytokines have generally occurred without pathway-specific loss of function, blockade, or rescue showing that the antiseizure or neuroprotective phenotype depends on the proposed inflammatory pathway. Recurrent reductions in these readouts therefore document an association with favorable outcomes and may indicate a contributing process, but a claim that seizure suppression is “mediated by NLRP3 inhibition” goes beyond the available experiments.

Reverse causality further limits interpretation. Seizures and status epilepticus can themselves induce neuroinflammatory, oxidative, cellular-injury, and related downstream responses [37]. A treatment that first reduces seizure severity may therefore secondarily normalize some inflammatory markers. Association between lower seizure burden and reduced inflammation does not establish the direction of causation.

### 7.4. Glial and Neurovascular Responses: Cell-Specific Causality Is Missing

Treatment-associated changes in astrocytic, microglial, and neurovascular readouts are similarly recurrent. Exendin-4 pretreatment reduced kainate-associated BBB leakage together with reactive-glial and inflammatory changes, while liraglutide, exendin-4, and the dual GLP-1R/GIPR agonist DA3-CH altered glial marker profiles in other seizure or post-SE models [21,28,33,35]. These findings support an association between GLP-1R-related treatment and changes in glial and neurovascular responses to seizures.

These observations do not identify a particular glial population as the mediator of seizure reduction or neuronal protection. Receptor-expression changes, immunostaining, and conventional reactive-state markers are insufficient to demonstrate a requirement for neuronal, astrocytic, microglial, or endothelial GLP-1R signaling. Reduced BBB leakage is similarly ambiguous with respect to causal order: barrier preservation could contribute to the seizure phenotype, but it could also follow reduced seizure severity or broader attenuation of tissue injury. Simplified microglial or astrocytic polarization categories provide little additional causal resolution. Cell-restricted GLP-1R or downstream pathway manipulation linked to epilepsy-relevant outcomes remains absent from the included studies.

The same receptor-specific caution applies to DA3-CH: because it activates both GLP-1R and GIPR, treatment-associated glial or neuronal effects cannot be assigned uniquely to GLP-1R without receptor dissection.

### 7.5. Oxidative, Mitochondrial, and Cell-Survival Pathways: Marker Changes Are Not Functional Rescue

Redox-, apoptosis-, autophagy-, and mitochondrial-dynamics-associated findings constitute another large component of the literature. GLP-1R-related treatments have been accompanied by changes in oxidative-stress markers, Bax/Bcl-2 and caspase-associated measures, LC3, and proteins related to mitochondrial dynamics or quality control in acute, kindling, Dravet, and post-SE models [20,25,30,34,35].

These measurements are compatible with reduced cellular stress or injury, but they should not be equated with restored mitochondrial function, normalized mitophagy, or rescued bioenergetics. Protein abundance or redox-marker normalization does not establish functional mitochondrial recovery, and pathway necessity has generally not been tested. The same reverse-causality problem applies: seizure attenuation itself may reduce oxidative stress, apoptotic signaling, and mitochondrial disturbance that can accompany seizure activity [37].

Overall, causal resolution remains uneven: receptor-level perturbation provides stronger evidence than the predominantly associative downstream pathway data. Neither a unified mediator of the antiseizure or neuroprotective effects nor an epilepsy-specific causal cell type has been established.

## 8. Semaglutide, CNS Exposure, and Next-Generation Incretin Therapies

Semaglutide combines unusually high translational relevance with an epilepsy-specific evidence base that is much narrower than the literature on liraglutide, exendin-4, and other GLP-1R-related interventions. Systemically administered GLP-1R agonists can influence CNS-associated biology, but such effects do not resolve exposure within epilepsy-relevant brain parenchyma or identify the route responsible for seizure-related phenotypes. For semaglutide in particular, clinical prominence, systemic pharmacokinetics, CNS exposure, target engagement, and epilepsy efficacy remain separate evidentiary questions.

### 8.1. Semaglutide: High Translational Relevance, Limited Direct Epilepsy Evidence

Epilepsy-specific evidence for semaglutide is currently concentrated in a single PTZ-kindling study. Treatment before and during repeated PTZ exposure reduced selected behavioral seizure scores and the proportion of fully kindled animals, increased seizure latency, and shortened the final provoked seizure duration [19]. The study supports direct semaglutide anti-kindling activity during concurrent treatment; it does not address efficacy in established chronic epilepsy, prevention of epilepsy, or durable disease modification. Its inflammatory and injury-related findings also remain associative rather than defining a semaglutide-specific causal pathway. Experimental dose alone does not establish clinically achievable exposure because the epilepsy studies reviewed here generally lack linked drug-specific systemic pharmacokinetics and regional brain-exposure measurements at doses associated with antiseizure effects.

Evidence from other GLP-1R-related compounds can inform biological plausibility but cannot substitute for direct semaglutide evidence. Liraglutide, exendin-4, native GLP-1, and engineered analogues differ in molecular structure, albumin binding, pharmacokinetics, tissue distribution, and experimental exposure [5,36]. Their epilepsy findings therefore cannot be transferred directly to semaglutide. Semaglutide remains a clinically prominent but comparatively under-tested epilepsy candidate rather than the best-established representative of a proven antiseizure class.

### 8.2. CNS Exposure: Distinguishing Access from Target Engagement

The CNS-access question is similarly more nuanced than a binary distinction between compounds that do or do not “cross the BBB.” Human evidence now shows that semaglutide can be detected in CSF after systemic treatment. In a small mechanistic study in individuals with early Alzheimer’s disease, low semaglutide concentrations were measurable in CSF after 12 weeks of once-weekly subcutaneous treatment [38]. Earlier human data likewise demonstrated very low CSF concentrations of liraglutide during chronic treatment [39]. Importantly, the liraglutide investigators explicitly noted that CSF concentrations could not be assumed to represent concentrations in relevant brain-parenchymal compartments.

Systemic exposure, CSF detection, brain-parenchymal or interstitial exposure, and GLP-1R occupancy represent different pharmacological measurements. Detecting a systemically administered drug in CSF establishes access to that compartment, but provides no direct measure of its concentration in hippocampus or cortex, extracellular availability around epilepsy-relevant cells, or receptor occupancy.

Preclinical semaglutide distribution studies reinforce this compartment-specific interpretation. In rodents, fluorescently labeled semaglutide accessed selected circumventricular, ventricular-adjacent, hypothalamic, and brainstem regions and induced neural responses in a broader distributed network, including regions without evidence of direct drug access [40]. Quantitative rat studies subsequently found very low overall brain-to-plasma partitioning, with nonuniform distribution and relatively higher concentrations in the hypothalamus [41]. Together, these findings are more consistent with restricted and regionally heterogeneous access than with free equilibration across an intact BBB.

**Figure 2:**
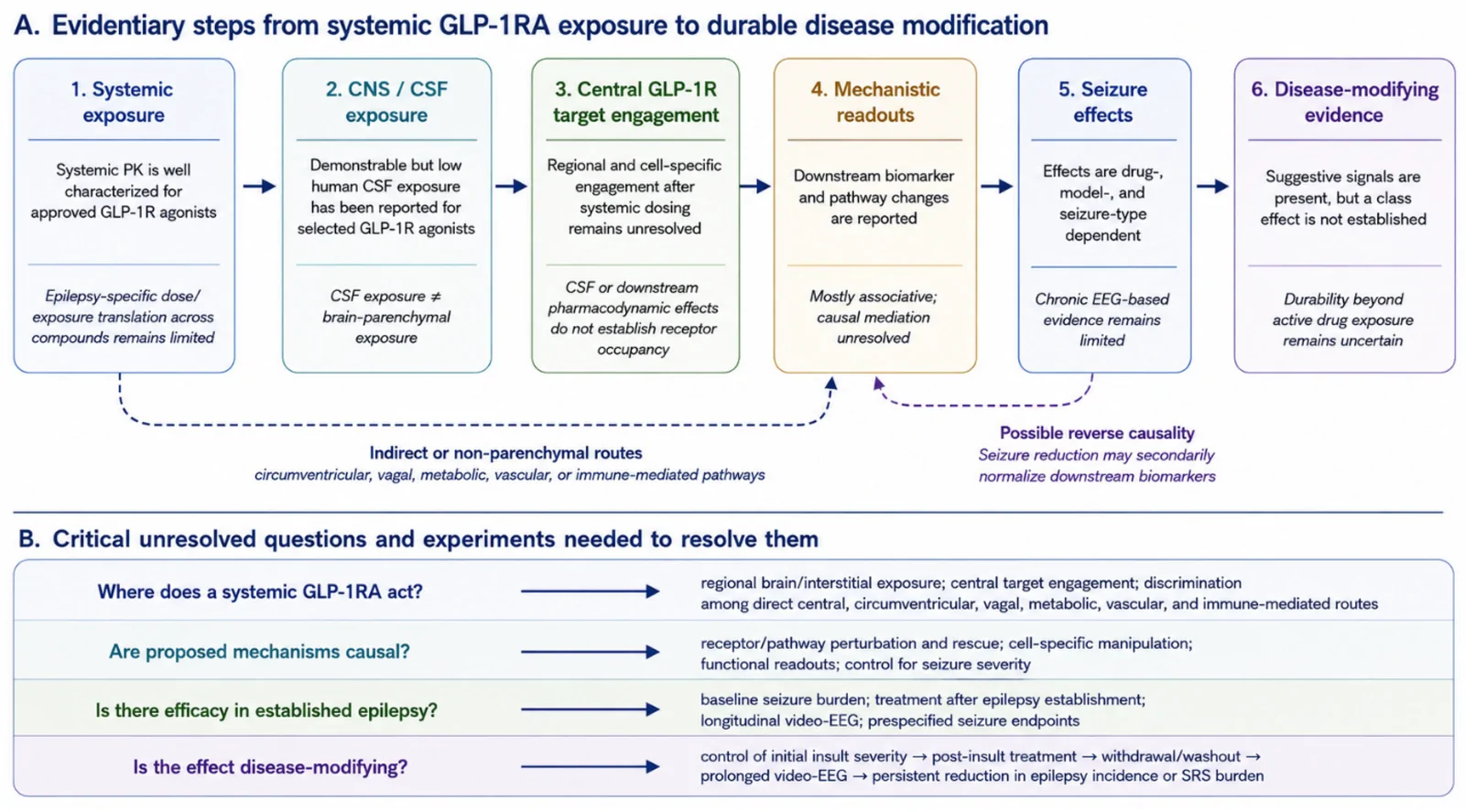
Evidentiary steps and critical gaps linking systemic GLP-1 receptor agonist exposure to disease-modifying effects in epilepsy. (**A**) Systemic drug exposure, CNS/CSF exposure, central GLP-1R target engagement, mechanistic readouts, seizure effects, and disease-modifying evidence represent distinct inferential steps and should not be treated as interchangeable. Low but demonstrable human CSF exposure has been reported for selected GLP-1R agonists; however, CSF detection does not establish brain-parenchymal exposure, regional receptor occupancy, or cell-specific target engagement. Moreover, a direct parenchymal route is not necessarily required for all CNS-related effects, because systemic GLP-1R agonists may also act through circumventricular, vagal, metabolic, vascular, or immune-mediated pathways. Most epilepsy-specific inflammatory, glial, oxidative, mitochondrial-associated, and neurotransmitter-related findings remain associative. The dashed reverse arrow indicates potential reverse causality, whereby reduced seizure burden may secondarily normalize downstream biomarkers. Antiseizure effects remain drug-, model-, and seizure-type-dependent, whereas evidence for durable disease modification is currently suggestive rather than established. (**B**) Key unresolved questions and experimental requirements for stronger causal and translational inference. These include regional brain/interstitial exposure and central target engagement studies; receptor- or pathway-level perturbation, rescue, and cell-specific manipulation; longitudinal video-EEG studies initiated after epilepsy is established; and disease-modification designs incorporating control of initial insult severity, post-insult treatment, treatment withdrawal or washout, prolonged monitoring of spontaneous recurrent seizures, and demonstration of persistent effects beyond active drug exposure.

Figure 2A summarizes the full inferential chain from systemic exposure to CNS/CSF exposure, central GLP-1R target engagement, downstream biological response, seizure outcome, and ultimately disease-modifying evidence. These steps must be resolved separately, while direct parenchymal entry is not the only plausible route. Circumventricular access, vagal or other neural afferents, systemic metabolic effects, vascular signaling, and peripheral immune modulation could all contribute to CNS-associated phenotypes [7,36]. The existence of a neurological or molecular response therefore does not identify which route is responsible.

Seizure- and status epilepticus-associated BBB disruption adds another layer of uncertainty because barrier integrity can be altered in epilepsy [42]. Within the closed epilepsy-specific evidence set, however, no pharmacokinetic study demonstrates that such disruption materially increases semaglutide or another GLP-1RA exposure in brain interstitial tissue. Enhanced access is therefore biologically plausible under conditions of BBB disruption, but pharmacologically meaningful exposure in epilepsy-relevant brain parenchyma has not been demonstrated.

### 8.3. Dual/Multi-Receptor Agonists and Adjacent Incretin Therapies

The pharmacological landscape is further broadened by dual and multi-receptor agonists. DA3-CH, for example, has shown treatment-associated post-SE effects [35], but because it activates both GLP-1R and GIPR, those effects cannot be assigned specifically to GLP-1R without receptor-level dissection. The same interpretive problem applies to clinically prominent dual agonists such as tirzepatide [5]: translational relevance does not establish that any seizure-related effect reproduces selective GLP-1R pharmacology.

More generally, increasing receptor multiplicity may broaden therapeutic possibilities while simultaneously weakening receptor-specific mechanistic attribution. DPP-4 inhibitors occupy an adjacent but distinct position: by altering endogenous incretin signaling they may inform the broader incretin–seizure relationship, but they are not direct GLP-1R agonists and should not be used as equivalent pharmacological evidence [18].

Overall, semaglutide and next-generation incretin therapies remain translationally relevant but require drug- and receptor-specific epilepsy studies rather than inference from systemic exposure, other agonists, or downstream CNS-associated effects.

## 9. Clinical and Translational Perspective

Human evidence provides important translational context but remains qualitatively different from therapeutic epilepsy evidence. Population-level studies generally do not show an increased coded seizure risk and several report inverse associations between GLP-1R-related exposure and seizure-related outcomes, yet most rely on observational designs, coded diagnoses, or adverse-event reporting rather than prospectively defined neurological efficacy endpoints. Table 3 therefore spans population-level epidemiological associations, limited observational evidence in people with established epilepsy, and broader randomized-exposure or post-marketing safety data. These findings do not establish antiseizure efficacy or a class-wide absence of seizure risk, and individual adverse and pharmacovigilance signals remain relevant.

### 9.1. Population-Level Observational Signals

Several large observational studies report inverse associations between GLP-1R-related treatment and incident seizure or epilepsy outcomes in populations with type 2 diabetes. In an active-comparator new-user analysis, GLP-1RA initiation was associated with a lower incidence of coded epilepsy than DPP-4 inhibition [12]. Agent-specific estimates were heterogeneous: semaglutide showed a stronger inverse association, whereas liraglutide and dulaglutide did not show comparably clear reductions. This heterogeneity argues against interpreting the overall association as a uniform GLP-1RA effect.

A separate target-trial emulation also found lower coded adult-onset seizure incidence after semaglutide initiation relative to selected glucose-lowering comparators [43]. De Giorgi et al. found no increased short-term coded seizure/epilepsy risk with semaglutide, providing primarily reassuring neurological safety evidence, whereas Xie et al. reported a more modest inverse seizure association that varied with comparator selection [13,44]. Together, these findings support an epidemiological signal rather than therapeutic efficacy. Propensity matching and active-comparator designs improve comparability but do not eliminate residual confounding related to treatment indication, metabolic disease severity, healthcare utilization, concomitant medication patterns, or other unmeasured factors. Improvements in glycemic control, weight, vascular risk, or systemic metabolic status may also contribute to the observed associations and cannot presently be disentangled from a direct CNS effect. The predominance of metabolically selected human cohorts also differs from many preclinical epilepsy models, and systemic metabolic effects may therefore contribute to the observational associations.

### 9.2. Evidence in Established Epilepsy

Observational evidence in people with established epilepsy is more directly relevant to the therapeutic question. AbuAlrob et al. reported lower coded seizure recurrence and status epilepticus among individuals receiving incretin-based therapies [45]. However, exposure was pharmacologically heterogeneous and included tirzepatide, so the association cannot be attributed specifically to selective GLP-1R activation. Outcomes were identified from electronic health records rather than prospective seizure diaries, adjudicated seizure counts, or electrographic monitoring, and residual confounding remains possible.

Human evidence is also not uniformly favorable. Findeis et al. described seizure exacerbation with semaglutide in an individual with structural epilepsy, with recurrence after re-exposure [46]. Positive rechallenge increases the plausibility of an individual semaglutide-associated adverse effect, but concomitant antiseizure-medication changes complicated causal attribution, and the single-patient design prevents definitive attribution to semaglutide or estimation of population risk.

Within the literature identified for this review through 6 August 2026, no randomized therapeutic epilepsy trial tested a GLP-1R agonist using prospectively defined seizure frequency or electrographic seizure burden as an efficacy endpoint. Semaglutide-specific observational associations must therefore remain distinct from evidence that semaglutide treats established epilepsy.

### 9.3. Randomized and Post-Marketing Safety Evidence

Randomized-exposure data are informative mainly for safety. A meta-analysis of placebo-controlled cardiovascular and renal trials found no excess seizure/epilepsy adverse-event signal with GLP-1R agonists and was compatible with fewer combined events in the GLP-1RA subgroup [47]. These rare, non-prespecified adverse events should not be interpreted as a randomized test of antiseizure efficacy.

Post-marketing pharmacovigilance provides a different type of safety evidence. Seizure-related reports have been identified after GLP-1RA exposure, including events associated with hypo- or hyperglycemia [48]. Spontaneous-report databases lack reliable denominators and are vulnerable to reporting bias and metabolic confounding, so these findings remain hypothesis-generating. Separately, GLP-1RA-induced slowing of gastric emptying can alter the absorption kinetics of some orally administered drugs, although clinically meaningful changes in overall exposure were generally not demonstrated in the oral-drug studies reviewed by Calvarysky et al. [49]. Within the literature reviewed through 6 August 2026, consequences for antiseizure-medication pharmacokinetics remained insufficiently characterized in epilepsy-specific studies.

Current human data comprise several inverse observational associations and safety-oriented findings for selected GLP-1R-related therapies, including semaglutide, but remain observational and pharmacologically heterogeneous. The established epilepsy signal relies on coded outcomes and nonrandomized exposure, whereas randomized-exposure and post-marketing data are more informative about safety than therapeutic efficacy. Individual adverse signals also argue against assuming uniform benefit. Human findings are directionally compatible with parts of the preclinical convulsive-model literature, but they do not resolve seizure-type specificity, mechanism, or disease modification. At present, they support translational plausibility rather than established antiseizure efficacy or modification of the epilepsy course.

## 10. Research Agenda: Experiments Needed to Resolve the Key Evidence Gaps

Figure 2B frames the remaining translational problem around four connected questions: where and through what route systemic GLP-1R-related therapies act, which downstream mechanisms are causal, whether they are effective after epilepsy is established, and whether any benefit persists as disease modification. Progress on these questions will depend less on additional acute seizure studies with expanding biomarker panels than on coordinated experiments that distinguish drug exposure, mechanism, seizure suppression, neuroprotection, and altered disease course.

### 10.1. Define the Route and Site of Action

One unresolved question is whether seizure-related effects require direct exposure of epilepsy-relevant brain regions or can arise through indirect neural, vascular, metabolic, or immune pathways. Addressing it will require systemic pharmacokinetics to be integrated with parallel measurements in plasma, CSF where informative, and relevant brain regions, ideally including interstitial or extracellular exposure where technically feasible. Regional exposure should be related temporally to both seizure effects and measures of GLP-1R target engagement.

This question is particularly important under conditions of seizure- or status epilepticus-associated BBB disruption. Rather than assuming that barrier impairment increases drug delivery, experiments should directly compare CNS exposure under intact and disrupted BBB conditions and determine whether any increase occurs at sites relevant to the seizure phenotype. However, pharmacokinetics, regional exposure, and target-engagement measurements can establish exposure and pharmacodynamic association but cannot by themselves demonstrate that a particular site or route is necessary for the effect. Where route or site necessity is the question, complementary route-discriminating or site-selective perturbation is required where feasible, for example by selectively manipulating receptor signaling within a candidate compartment or interrupting a candidate neural route. Such experiments should discriminate among direct parenchymal, circumventricular, neural, vascular, metabolic, and immune-mediated actions without presuming which pathway is dominant.

### 10.2. Move from Biomarker Association to Causal Mechanisms

Mechanistic studies should likewise move beyond treatment-associated molecular changes. Candidate pathways can be tested by combining receptor-level manipulation with pathway-specific perturbation and, where possible, rescue. A persuasive causal sequence would show both that treatment modifies the candidate pathway and that selective disruption of that pathway alters or abolishes the seizure or neuroprotective effect.

Cell-specific causality represents a particularly important gap. Conditional receptor deletion, knockdown, rescue, or targeted downstream manipulation in defined neural, glial, or neurovascular populations could determine which cellular compartments are necessary. Such studies should also address reverse causality, because reduced seizure burden can itself normalize inflammatory, glial, oxidative, apoptotic, and mitochondrial-associated readouts.

### 10.3. Test Efficacy After Epilepsy Is Established

Studies intended to test efficacy in established epilepsy should begin treatment only after the epileptic phenotype has been documented. A core design would establish a pretreatment seizure baseline, randomize animals after that baseline, initiate treatment after epilepsy is established, and use longitudinal video-EEG to quantify seizure frequency, duration, and burden.

This design is fundamentally different from acute pretreatment, concurrent kindling, or immediate post-SE intervention. Seizure-type specificity should also be tested rather than inferred from convulsive models, particularly given the discordant absence-epilepsy findings. Semaglutide is a high-priority translational test case because of its clinical prominence, but it should not be treated as a proxy for the GLP-1RA class.

### 10.4. Demonstrate Durable Disease Modification

Future studies designed to provide a persuasive test of disease modification should incorporate additional disease-course controls. In post-insult paradigms, the severity of the initiating insult should be documented and balanced or analytically controlled. Longitudinal SRS or epilepsy-incidence endpoints should then be followed through treatment and into an off-treatment period.

Demonstrating persistence beyond active exposure would provide particularly strong evidence for separating disease modification from continued antiseizure action. Withdrawal should therefore be interpreted relative to the pharmacokinetics of the specific compound. Parallel designs are needed for two distinct questions: whether post-insult treatment prevents or delays epilepsy development, and whether treatment of established epilepsy produces a persistent change in seizure burden after withdrawal.

The same principles should guide early clinical translation: prospective trials should prioritize randomized exposure, documented baseline seizure frequency, prespecified seizure outcomes, monitoring for seizure worsening, and clinically relevant antiseizure-medication pharmacokinetics. Biomarkers and metabolic changes should remain secondary measures rather than substitutes for therapeutic seizure endpoints.

These questions are best addressed as a linked experimental program rather than as a single omnibus study. Chronic epilepsy experiments with documented baseline seizure burden, longitudinal EEG, appropriate treatment timing, and withdrawal can provide the core efficacy and disease-course framework. Complementary modules can then resolve drug-specific systemic and regional exposure, target engagement, route discrimination, pathway necessity, and cell-specific causality. Such a program would be more informative than additional biomarker-rich acute studies and would directly separate exposure-dependent antiseizure activity from neuroprotection and genuine disease modification.

## 11. Conclusions

Selected GLP-1R-related interventions show direct antiseizure and anti-kindling activity, but the effects vary with compound, model, treatment timing, and seizure type. Much of the positive evidence comes from acute pretreatment or concurrent-treatment paradigms, while data from established chronic epilepsy remain limited. Null and pro-seizure findings in absence-epilepsy models provide important counterevidence to a uniform class-wide antiseizure effect. Neuroprotective findings extend across a broader set of paradigms and include direct neuronal or tissue preservation, although much of the literature still relies on molecular or inflammatory surrogates. Because lower seizure severity can itself reduce downstream injury, biomarker normalization is not equivalent to direct neuroprotection; even direct neuronal preservation does not by itself demonstrate disease modification. Concurrent attenuation of kindling likewise remains evidence of anti-kindling activity rather than demonstrated antiepileptogenesis.

Evidence for disease modification is still suggestive. Selected chronic/SRS studies and limited persistence findings are compatible with altered seizure burden or disease development, but uncertainties in baseline seizure characterization, longitudinal EEG sampling, control of the initiating insult, and persistence after treatment withdrawal prevent definitive attribution to durable modification of epilepsy. Mechanistic evidence shows a similar hierarchy. Receptor loss, antagonism, and rescue provide meaningful causal leverage for GLP-1R involvement in selected paradigms, whereas proposed synaptic, inflammatory, glial, oxidative, mitochondrial, and cell-survival pathways remain predominantly associative. Neither a unified downstream causal mechanism nor an epilepsy-specific causal cell type has been established.

Pharmacological heterogeneity further limits extrapolation across GLP-1R-related interventions. Semaglutide has high translational relevance but remains directly under-tested in epilepsy, and evidence from other agonists cannot substitute for semaglutide-specific data. Emerging human evidence comprises several inverse observational associations and safety-oriented findings for selected therapies, while individual adverse signals and agent heterogeneity argue against assuming uniform benefit. Within the literature identified for this review through 6 August 2026, no randomized therapeutic epilepsy trial tested GLP-1R agonists using prospectively defined seizure-frequency or electrographic efficacy endpoints. The current evidence justifies more decisive translational testing, not claims of established clinical antiseizure efficacy or disease modification in epilepsy.

## Figures and Tables

**Table 1:** Direct preclinical evidence for GLP-1R-related interventions in seizure and epilepsy models.

Intervention	Model/Design	Antiseizure Outcome	Neuroprotective Outcome	Disease-Modifying Inference	Key Interpretive Consideration	Ref.
**I. Chronic, spontaneous, or genetic seizure models**						
L 300 µg/kg/d s.c.	KA-SRS mouse: +6 h → 28 d; WAG/Rij rat: P30 → 17 wk + 30 d WD	KA: ↓SRS, ↓cumulative duration (vEEG); WAG/Rij: 0 SWD prevention	KA: partial CA1 preservation	KA: suggestive; WAG/Rij: no prevention	No baseline seizure burden; KA EEG was sampled for 3 h/day on 2 days, and off-treatment persistence in the KA arm was assessed only briefly.	[11]
L 150 µg/kg/d i.p.; 14 d	*Scn1a*+/− Dravet mouse	Reported ↓seizure susceptibility/EEG abnormalities	Reported neuronal preservation; ↓apoptotic markers	Post-Tx persistence only hypothesis-generating	Later testing provides a possible off-treatment signal, but quantitative longitudinal seizure assessment is limited.	[20]
E4 20 µg/kg/d i.p.; 21 d	Pilo-SE → SRS mouse; post-SE Tx	Reported ↓SRS frequency and duration	↓apoptosis and reactive-glial/inflammatory markers	NE	Post-SE seizure monitoring was limited; cell-specific GLP-1R dependence was not tested.	[21]
E4 10/50/100 µg/kg i.p.; single dose	WAG/Rij rat; established absence epilepsy; 180 min ECoG	↑SWD number and duration; amplitude 0	—	—	Single-dose, 180 min ECoG study; potential effects of vigilance/behavioral state on SWDs were not separately assessed.	[15]
L 75–300 µg/kg/d s.c. ± DZP; acute or 10 wk	GEPR-9 audiogenic rat; chronic arm + 4-wk WD	Minimal acute effect; modest chronic benefit; preserved DZP efficacy/↓tolerance	—	—	Seizure outcomes were behaviorally assessed; chronic effects were not evaluated with EEG, and epilepsy prevention was not tested.	[22]
**II. Kindling and repeated-provocation models**						
S 10/25 nmol/kg i.p., EOD	PTZ-kindling mouse; treatment began 8 d before kindling and continued during acquisition; final PTZ/ECoG challenge after additional S dose	↓selected scores/full-kindling rate; ↑latency; ↓final seizure duration	↓hippocampal injury markers; improved cognition	NE *	Kindling and the final PTZ/ECoG challenge were assessed under active semaglutide exposure; no off-treatment seizure assessment was performed; quantitative ECoG was limited, and NLRP3 mediation was not directly tested.	[19]
L 0.5 mg/kg/d s.c. ± E9 0.1 mg/kg i.c.v.	PTZ-kindled rat; challenge ≈4 d off Tx	Delayed severe kindling; ↓severe episodes/mean score; E9 reversed effect	—	Suggestive anti-kindling; not epilepsy prevention	Short post-treatment PTZ/EEG challenge remained a provoked assessment; persistence was evaluated only over a brief interval, without prolonged off-treatment follow-up.	[10]
L 75/150 µg/kg/d i.p.	PTZ-kindling mouse; concurrent Tx	↓behavioral seizure severity during kindling/challenge	Biochemical changes only	NE *	Behavioral kindling study without EEG or withdrawal assessment; no agonist-only comparison.	[23]
L 75/150 µg/kg/d i.p.	Corneal electrical kindling mouse; concurrent Tx	↓behavioral seizure severity during kindling/challenge	Biochemical changes only	NE *	Behavioral kindling study without after-discharge EEG or withdrawal assessment.	[24]
L 75 µg/kg/d i.p.; 2 wk	PTZ-kindling rat; concurrent Tx	↓Racine score; ↑latency; duration 0	Marker-based support only	NE *	Behavioral kindling outcomes without EEG or withdrawal assessment; molecular findings were based on selected tissue endpoints.	[25]
L 75/150 µg/kg i.p. ± LEV 50 mg/kg; concurrent	PTZ-kindling mouse	Delayed stage-5 kindling; no seizure advantage over LEV	Behavioral/biochemical associations only	NE *	Concurrent kindling treatment without EEG or withdrawal assessment; the design demonstrates delayed kindling rather than prevention of epilepsy.	[26]
**III. Acute seizure and receptor-pathway studies**						
GLP-1R KO/rescue; [Ser(2)]exendin(1–9)	Acute KA mouse; WT/Het/KO + hippocampal rescue	KO worsened seizures; Het intermediate; rescue improved phenotype	Receptor-dependent ↓KA neuronal injury	—	Acute KA model without chronic disease-course assessment; the experimental peptide is not a clinically used GLP-1RA.	[9]
GLP-1 1–1000 ng i.c.v.; pre-Pilo	Acute pilocarpine-SE rat	0 effect on SE incidence, latency, or severity	—	—	Acute behavioral seizure study without EEG or chronic follow-up.	[27]
E4 200 nmol/kg i.p.; −72 h	Acute KA mouse; pretreatment	↓acute seizure score	↓neuronal death, inflammatory markers, BBB leakage	—	Acute pretreatment design without EEG; injury reduction may partly follow reduced acute seizure severity; GLP-1R dependence was not directly tested.	[28]
EX 50/100/200 µg/kg i.p.; acute	Penicillin cortical spikes rat; 3 h ECoG	200: ↓spike frequency in selected intervals; latency/amplitude 0	—	—	Acute 3 h ECoG experiment under anesthesia; no chronic seizure or disease-course outcomes.	[29]
L 3/6 mg/kg i.p.; −30 min	Acute PTZ rat; separate EEG/behavior cohorts	↓EEG activity/Racine score; ↑myoclonic-jerk latency	—	—	Acute pretreatment study with separate EEG and behavioral cohorts; no chronic or off-treatment outcomes.	[30]
L 100/200 µg/kg i.p.; −30 min	Acute picrotoxin convulsions mouse	Delayed onset; ↓tonic-convulsion duration; mortality lacked dose–response	—	—	Brief acute pretreatment study with a 15 min observation period; no EEG or chronic outcomes.	[31]
E9 10 mg/kg i.p. in vivo; GLP-1R siRNA in cultured neurons	Hyperthermia, febrile seizures, P21 rat + cultured hippocampal neurons	In vivo E9: ↑seizure severity, ↑stage-4 bursts, ↑EEG amplitude, ↓seizure latency	—	—	In vivo seizure/EEG effects were tested with E9; GLP-1R siRNA was used only in cultured neurons for synaptic/TRPV1 experiments. No agonist was tested.	[32]
**IV. Early post-SE tissue studies without post-treatment seizure outcomes**						
L 25 nmol/kg/d i.p.; 0 → 7 d post-SE	Li–Pilo SE rat; early tissue study	—	Early inflammatory/apoptosis-marker shifts; neuronal protection not directly shown	NE; no post-Tx seizure outcome	Early post-SE tissue study without a post-treatment seizure endpoint or direct neuronal-survival assessment; no chronic epilepsy assessment.	[33]
L 300 µg/kg/d i.p.; +3 h × 3 d	Li–Pilo SE rat; early post-SE	—	Selected redox/inflammatory/mitochondrial markers improved; weak cognitive protection	NE; no post-Tx seizure outcome	Short early post-SE treatment; no post-treatment seizure endpoint; mechanistic conclusions rely predominantly on molecular markers.	[34]
D3 10 nmol/kg/d i.p.; +12 h → 7 d	Li–Pilo SE rat; after DZP-terminated SE	—	↓GFAP/Iba1, IL-1β/TNF, Bax; ↑Bcl-2/NeuN preservation	NE; no chronic seizure outcome	Seven-day post-SE follow-up without chronic seizure outcomes; equivalence of initial SE severity between groups was not demonstrated.	[35]

**Symbols:** ↓, decrease; ↑, increase; 0, no significant effect; —, outcome not assessed/not applicable; NE, disease modification not established. **Interpretive note:** Entries in the “Key interpretive consideration” column identify design features relevant to the scope of inference and are not intended as a formal risk-of-bias or study-quality assessment. * Concurrent treatment during kindling/repeated provocation does not by itself establish antiepileptogenesis or disease modification. **Abbreviations:** BBB, blood–brain barrier; D3, DA3-CH (dual GLP-1R/GIPR agonist); DZP, diazepam; E4, exendin-4; E9, exendin(9–39); ECoG, electrocorticography; EOD, every other day; EX, exenatide; GIPR, glucose-dependent insulinotropic polypeptide receptor; GLP-1R, glucagon-like peptide-1 receptor; KA, kainic acid; L, liraglutide; LEV, levetiracetam; Pilo, pilocarpine; PTZ, pentylenetetrazole; S, semaglutide; SE, status epilepticus; SRS, spontaneous recurrent seizures; SWD, spike–wave discharge; Tx, treatment; vEEG, video-EEG; WD, withdrawal. Shaded rows indicate model/design categories.

**Table 2:** Mechanistic evidence for GLP-1R-related effects in seizure and epilepsy models: association versus causality.

Mechanistic Domain	Key Evidence in Seizure/Epilepsy Models	Causal Interpretation	Ref.
GLP-1R dependence	*Glp1r* loss worsened acute KA seizures and neuronal injury, whereas hippocampal rescue improved the phenotype; exendin(9–39) reversed key liraglutide anti-kindling effects.	Receptor-dependent in these paradigms. GLP-1R signaling limits acute KA seizure susceptibility and contributes to liraglutide anti-kindling effects in PTZ kindling; this does not establish a uniform agonist class effect or identify the downstream mediator(s).	[9,10]
Synaptic E/I regulation	GLP-1R inhibition or knockdown reduced GABAergic sIPSCs in cultured hippocampal neurons under febrile-seizure-related conditions; liraglutide altered GABA_A_-, AMPA-, and NMDA-receptor profiles in PTZ kindling.	Functional support for inhibitory synaptic regulation; broader E/I rebalancing remains unproven. TRPV1 necessity and the functional consequences of receptor-expression changes have not been established in vivo.	[10,32]
Neuroinflammatory/NLRP3-related signaling	Across several GLP-1R-related interventions, reductions in NLRP3- and cytokine-associated readouts were reported in PTZ-kindling and post-SE studies.	Associative. The anti-inflammatory signal is recurrent, but no pathway loss-of-function, rescue, or blockade demonstrates mediation of seizure or neuroprotective effects.	[19,21,34,35]
Glial/neurovascular responses	Exendin-4, liraglutide, and DA3-CH altered reactive-glia markers; exendin-4 pretreatment reduced KA-associated BBB leakage.	Associative. Changes in reactive-glia markers and reduced BBB leakage are supported as treatment-associated outcomes, but cell-specific GLP-1R causality and whether barrier preservation mediates seizure or neuroprotective effects remain unestablished.	[21,28,33,35]
Oxidative/mitochondrial/cell-survival signaling	Redox, mitochondrial-dynamics, and apoptosis-related markers shifted with treatment in acute, kindling, Dravet, and post-SE models.	Associative. Injury-related marker shifts are compatible with neuroprotection but do not demonstrate restored mitochondrial function or pathway necessity; some changes may also be secondary to reduced seizure burden.	[20,25,30,34,35]

**Interpretive note:** Mechanistic interpretation reflects the experimental leverage supporting a specific causal claim and is not an overall study-quality assessment. For the purposes of this review, marker abundance, cytokine levels, bulk neurotransmitter concentrations, or protein-expression changes without functional or interventional testing are treated as associations. The table highlights representative recurrent or causally informative mechanistic domains and is not intended as an exhaustive catalogue of all reported molecular changes. **Cell-specific causality:** For the purposes of this review, cell-specific causality would require cell-restricted GLP-1R or pathway manipulation linked to seizure or neuroprotective outcomes; this has not been established in the included seizure/epilepsy studies. DA3-CH is a dual GLP-1R/GIPR agonist; its effects cannot be attributed specifically to GLP-1R. **Abbreviations:** BBB, blood–brain barrier; E/I, excitation/inhibition; GIPR, glucose-dependent insulinotropic polypeptide receptor; GLP-1R, glucagon-like peptide-1 receptor; KA, kainic acid; PTZ, pentylenetetrazole; SE, status epilepticus; sIPSC, spontaneous inhibitory postsynaptic current.

**Table 3:** Human evidence relevant to seizure and epilepsy outcomes with GLP-1R-related therapies.

Design/Population/Exposure	Seizure-Related Outcome/Ascertainment	Main Finding	Maximum Justified Inference	Ref.
**I. Population-level observational evidence**				
Active-comparator new-user EHR cohort; adults with T2DM; 1:1 matched GLP-1RA vs. DPP-4i, *n* = 226,383/group (452,766 total)	New coded epilepsy diagnosis; no EEG or clinical adjudication	Lower coded incident epilepsy risk with GLP-1RA exposure, HR 0.84 (0.78–0.90); the inverse association was stronger for semaglutide, HR 0.68 (0.60–0.77), whereas liraglutide and dulaglutide did not show clear inverse associations.	Inverse observational association; does not establish epilepsy prevention or a uniform GLP-1RA class effect.	[12]
Target-trial emulation; adults with T2DM; semaglutide vs. other GLDs, *n* = 2586 vs. 7627, and vs. SGLT2i, *n* = 2814 vs. 5791	Coded incident adult-onset seizure; sensitivity definitions applied	Lower coded seizure risk versus selected comparators, with weighted HRs of 0.44 (0.25–0.79) and 0.48 (0.27–0.85).	Semaglutide-specific inverse epidemiological association; does not establish antiseizure efficacy, antiepileptogenesis, or direct CNS action.	[43]
Propensity-matched EHR analyses; adults with T2DM; three 1:1 comparisons: *n* = 23,386, 22,584, and 19,206 per arm vs. sitagliptin, empagliflozin, and glipizide, respectively	Coded seizure/epilepsy among multiple neurological outcomes; 12-month follow-up	No increased coded seizure/epilepsy risk was detected across comparator analyses.	Provides a reassuring short-term neurological safety signal; does not establish antiseizure efficacy.	[13]
Large observational cohorts of US Veterans with diabetes; 215,970 GLP-1RA initiators; separate comparator cohorts *n* = 117,989–1,203,097 depending on analysis	Coded seizures among 175 phenome-wide outcomes	Modest inverse association versus usual care, HR 0.90 (0.85–0.95); effect magnitude depended on comparator choice.	Exploratory population-level signal; comparator-dependent and not therapeutic evidence.	[44]
**II. Evidence in people with established epilepsy**				
EHR cohort; adults with established epilepsy, mostly with T2DM; pooled incretin therapies including tirzepatide; 8688 matched pairs (17,376 analyzed)	Coded seizure recurrence and status epilepticus; no EEG or seizure diary	Lower coded seizure recurrence, HR 0.82 (0.78–0.86), and status epilepticus, HR 0.75 (0.66–0.85).	Observational signal in established epilepsy; does not establish therapeutic antiseizure efficacy or a selective GLP-1R effect.	[45]
Single patient (*n* = 1) with structural epilepsy; semaglutide exposure with dechallenge/rechallenge	Clinical seizure cluster/focal motor status; dechallenge/rechallenge; concomitant ASM changes complicate interpretation	Seizure exacerbation recurred after semaglutide re-exposure.	Positive rechallenge supports an individual semaglutide-associated safety signal; population-level risk cannot be estimated.	[46]
**III. Randomized-exposure and post-marketing safety evidence**				
Meta-analysis of 27 placebo-controlled cardiovascular/renal RCTs (197,910 participants with outcome data); GLP-1RA subgroup: 8 RCTs, ~60,000 participants	Rare seizure/epilepsy adverse events; not prespecified neurological efficacy endpoints	No increased reported seizure/epilepsy adverse-event signal; the GLP-1RA subgroup was compatible with fewer combined events, RR 0.67 (0.46–0.98).	Randomized-exposure safety evidence, but not a randomized epilepsy efficacy test.	[47]
FAERS pharmacovigilance analysis, 2005 Q2–2024 Q3; six GLP-1R agonists; 250,014 GLP-1RA-associated AE reports overall, including 28,953 neurological AE reports; no exposed-population denominator	Spontaneously reported seizure-related adverse events	Detected seizure-related terms included hypoglycemic and hyperglycemic seizures, highlighting substantial metabolic confounding.	Hypothesis-generating safety signal only; incidence and causal risk cannot be inferred.	[48]

Interpretive note: Available human evidence is observational or safety-oriented. No included study was a randomized therapeutic epilepsy trial with seizure frequency or electrographic seizure burden as a prespecified efficacy endpoint. Coded diagnoses and adverse-event outcomes therefore should not be interpreted as proof of antiseizure efficacy, antiepileptogenesis, or disease modification. Effect estimates are shown with 95% confidence intervals in parentheses. Abbreviations: ASM, antiseizure medication; CNS, central nervous system; DPP-4i, dipeptidyl peptidase-4 inhibitor; EHR, electronic health record; FAERS, FDA Adverse Event Reporting System; GLP-1RA, GLP-1 receptor agonist; HR, hazard ratio; RCT, randomized controlled trial; RR, risk ratio; T2DM, type 2 diabetes mellitus. Shaded rows indicate evidence categories.

## Data Availability

No new data were created or analyzed in this study. Data sharing is not applicable to this article.
